# Microcirculatory alterations in critically ill COVID-19 patients analyzed using artificial intelligence

**DOI:** 10.1186/s13054-022-04190-y

**Published:** 2022-10-14

**Authors:** Matthias Peter Hilty, Emanuele Favaron, Pedro David Wendel Garcia, Yavuz Ahiska, Zuhre Uz, Sakir Akin, Moritz Flick, Sesmu Arbous, Daniel A. Hofmaenner, Bernd Saugel, Henrik Endeman, Reto Andreas Schuepbach, Can Ince

**Affiliations:** 1 Institute of Intensive Care Medicine, University Hospital of Zurich, Rämistrasse 100, 8091 Zurich, Switzerland; 2 Department of Intensive Care, Erasmus MC, University Medical Center, Rotterdam, The Netherlands; 3 Active Medical BV, Leiden, The Netherlands; 4 Department of Intensive Care, Leiden University Medical Center, Leiden, The Netherlands; 5 Department of Intensive Care, Haga Hospital, The Hague, The Netherlands; 6 Department of Anesthesiology, Center of Anesthesiology and Intensive Care Medicine, University Medical Center Hamburg-Eppendorf, Hamburg, Germany

**Keywords:** Microcirculation, COVID-19, Deep learning, Neuronal network, Artificial intelligence

## Abstract

**Background:**

The sublingual microcirculation presumably exhibits disease-specific changes in function and morphology. Algorithm-based quantification of functional microcirculatory hemodynamic variables in handheld vital microscopy (HVM) has recently allowed identification of hemodynamic alterations in the microcirculation associated with COVID-19. In the present study we hypothesized that supervised deep machine learning could be used to identify previously unknown microcirculatory alterations, and combination with algorithmically quantified functional variables increases the model’s performance to differentiate critically ill COVID-19 patients from healthy volunteers.

**Methods:**

Four international, multi-central cohorts of critically ill COVID-19 patients and healthy volunteers (*n* = 59/*n* = 40) were used for neuronal network training and internal validation, alongside quantification of functional microcirculatory hemodynamic variables. Independent verification of the models was performed in a second cohort (*n* = 25/*n* = 33).

**Results:**

Six thousand ninety-two image sequences in 157 individuals were included. Bootstrapped internal validation yielded AUROC(CI) for detection of COVID-19 status of 0.75 (0.69–0.79), 0.74 (0.69–0.79) and 0.84 (0.80–0.89) for the algorithm-based, deep learning-based and combined models. Individual model performance in external validation was 0.73 (0.71–0.76) and 0.61 (0.58–0.63). Combined neuronal network and algorithm-based identification yielded the highest externally validated AUROC of 0.75 (0.73–0.78) (*P* < 0.0001 versus internal validation and individual models).

**Conclusions:**

We successfully trained a deep learning-based model to differentiate critically ill COVID-19 patients from heathy volunteers in sublingual HVM image sequences. Internally validated, deep learning was superior to the algorithmic approach. However, combining the deep learning method with an algorithm-based approach to quantify the functional state of the microcirculation markedly increased the sensitivity and specificity as compared to either approach alone, and enabled successful external validation of the identification of the presence of microcirculatory alterations associated with COVID-19 status.

**Supplementary Information:**

The online version contains supplementary material available at 10.1186/s13054-022-04190-y.

## Introduction

Assessment of the sublingual microcirculation has been shown to display disease-specific functional alterations and provide insight into the success of resuscitation measures in critically ill patients [1, 2]. Recently developed algorithms such as MicroTools to automatically analyze handheld vital microscopy (HVM) image sequences of the sublingual microcirculation have introduced the possibility to quantify the determinants of hemoglobin transport to the tissue, making it possible to objectively measure microcirculatory function. Functional capillary density (FCD) [3] and capillary hematocrit (cHct) [2, 4, 5] as measures of microcirculatory diffusion capacity, and red blood cell velocity (RBCv) [3] as measure of microcirculatory convection capacity, have been shown to differentiate all relevant forms of circulatory shock from baseline, and differentiate the effects of interventions to recruit the microcirculation [1]. However, disease-specific functional alterations may include information besides the variables related to the oxygen delivery capacity of the microcirculation.

Neuronal networks are complex series of mathematical models mimicking the interplay of biological neurons in the central nervous system. When aimed at advanced image analysis [6], they are increasingly being adopted for their potential to complement dedicated algorithms, augmenting them with their ability to identify unknown features in decision support for the diagnosis of medical conditions [7]. The aim of the present study was to determine if deep learning using a convolutional neuronal network has the capacity to differentiate critically ill COVID-19 patients from healthy individuals by analysis of the sublingual microcirculation images better than conventional statistics such as the use of a logistic regression model employing algorithm-derived functional hemodynamic variables of the microcirculation. Our hypotheses were that (I) supervised training of a two-dimensional convolutional neuronal network with HVM image sequences of the sublingual microcirculation differentiates critically ill COVID-19 patients from healthy volunteers, and that (II) the combination of a two-dimensional convolutional neuronal network designed for recognition of COVID-19-associated microcirculatory alterations with algorithmically quantified microcirculatory hemodynamic variables provides an increased performance of the model as compared to either method alone.

## Methods

This study was performed using an international, multi-central dataset of critically ill COVID-19 patients treated in four tertiary intensive care units between March 2020 and June 2021, located in the University Hospital of Zurich, Switzerland, Erasmus Medical Center, Rotterdam, The Netherlands, the Haga Hospital, The Hague, The Netherlands, and the Leiden University Medical Center, Leiden, The Netherlands. The first measurements within this cohort have been described in an earlier study [2]. Two separate cohorts of healthy volunteers served as control groups [8]. Informed consent for study participation and publication of anonymized data was obtained from each subject prior to enrollment. The study was approved in the respective centers by the ethics committee of the University of Zurich (BASEC–ID2020–00,646), the Erasmus Medical Center medical ethics committee (MEC2018–1572), the ethics committee of the Hamburg Medical Association (PV5635), the Leiden University Medical Center (Coco2021–018), and the University of Bern (KEK–226/12). The study was conducted in accordance with the Declaration of Helsinki.

### Dataset and study design

**Fig. 1:**
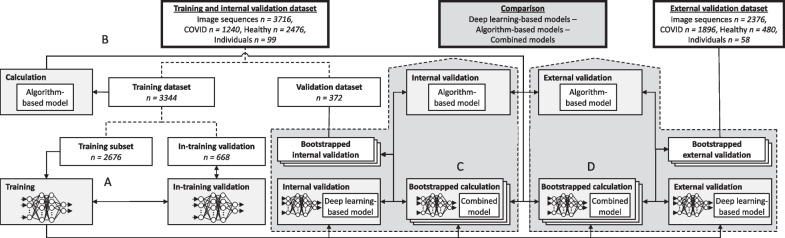
Sublingual handheld vital microscopy (HVM) image sequences recorded in four international, multi-central cohorts of critically ill COVID-19 patients and healthy volunteers were used for neuronal network training, model generation and internal validation, and two separate cohorts were used for external validation. Each dataset consisted of the neuronal network input matrix derived from the HVM image sequences, the functional microcirculatory variables derived from the image sequences by algorithm, and the reference COVID-19 disease state categorization. The training dataset was used to train the neuronal network, yielding the deep learning-based model (**A**), and to calculate the algorithm-based model (**B**). Both models were evaluated in the internal validation dataset in a bootstrapped process to identify the presence of microcirculatory alterations associated with COVID-19 disease state, and a combined model was calculated and internally validated in a separate, bootstrapped process (**C**). All models were then externally validated in an independent dataset (**D**). The results provided by all three models in the internal and external validation dataset were used to calculate the area under the receiver operating characteristic curve in a bootstrapped model, to quantify and compare their performance for identification of the presence of microcirculatory alterations associated with COVID-19 disease state in sublingual microcirculation HVM image sequences. HVM, handheld vital microscopy

Overall, sublingual microcirculatory measurements were taken in 84 critically ill COVID-19 patients and compared to 73 healthy volunteers. Inclusion criteria for the critically ill COVID-19 patients were a laboratory-confirmed SARS-CoV-2 infection by nucleic acid amplification according to the WHO-issued testing guidelines, and severe manifestation of COVID-19 requiring treatment in an ICU [9, 10]. The measurements from 59 COVID-19 patients treated in Zurich, Rotterdam and The Hague, and 40 healthy volunteers enrolled in Hamburg (Table 1), were used to train a convolutional neuronal network to differentiate critically ill COVID-19 patients from healthy volunteers after random assignment to the training or internal validation dataset with a ratio of 0.9/0.1 stratified by the disease state (Fig. 1A). Functional microcirculatory hemodynamic variables, namely FCD, cHct and RBCv, were additionally calculated from all image sequences using the MicroTools algorithm [3], and these were used to calculate a logistic regression model for the algorithm-based identification of the disease state (Fig. 1B). The internal validation dataset was then used to validate both the trained deep learning-based, and the algorithm-based model using a bootstrap process. A combined logistic regression model was then calculated based on the results from the deep learning-based and algorithm-based models in the internal validation dataset via an independent bootstrap process (Fig. 1C). Finally, to test the generalizability of the method, the deep learning methodology we developed was applied alongside the algorithm-based and combined models, to a completely new COVID-19 cohort consisting of 25 critically ill COVID-19 patients treated at Leiden University Medical Center, plus a new volunteer set 33 healthy volunteers measured in Zurich (Table 1, Fig. 1D). The bootstrapped area under the receiver operating characteristic curve (AUROC) distributions were used to compare the performance of all three model types, and the results obtained from the internal and external validation cohorts.

**Table 1:** Characteristics of the critically ill COVID-19 patients and healthy volunteers included in the present study

	Training and internal validation cohort			External validation cohort		
	COVID-19 patients (Zurich / Rotterdam/The Hague cohorts) *n* = 59	Healthy volunteers (Hamburg cohort) *n* = 40	*P* value	COVID-19 patients (Leiden cohort) *n* = 25	Healthy volunteers (Zurich cohort) *n* = 33	*P* value
*Characteristics at study inclusion*						
Age	59.5 ± 10.6	24.1 ± 1.8	< 0.0001	60.8 ± 10.7	45.8 ± 12.1	< 0.0001
Sex [male]	43/54 (80%)	17/40 (42.5)	< 0.0001	18/25 (72%)	15/33 (55%)	< 0.0001
Body mass index [kg m^−2^]	29.0 ± 6.0	22.8 ± 2.9	< 0.0001	30.0 ± 6.0	23.1 ± 4.5	< 0.0001
Duration of COVID symptoms before inclusion [days]	17 (12–27)	–	–	10 (9–11)	–	–
Days from ICU admission to inclusion [days]	7 (4–12)	–	–	2 (1–3)	–	–
*Physiological status at study inclusion*						
O_2_ saturation [%]	93 ± 3	–	–	92 ± 10	98 ± 1	0.0006
PaO_2_ [mmHg]	77 ± 24	–	–	84 ± 21	95 ± 8	< 0.0001
FiO_2_ [%]	48 ± 17	21 ± 0	< 0.0001	51 ± 18	21 ± 0	< 0.0001
PaO_2_/FiO_2_ ratio	178 ± 79	–	–	187 ± 67	454 ± 38	< 0.0001
PEEP [cmH2O]	15.7 ± 6.9	–	–	11.5 ± 4.1	–	–
pH	7.39 ± 0.08	–	–	7.39 ± 0.07	7.44 ± 0.02	< 0.0001
Lactate [mmol L^−1^]	1.2 ± 0.4	–	–	2.1 ± 0.7	0.8 ± 0.2	< 0.0001
Hemoglobin [g L^−1^]	97 ± 18	–	–	127 ± 14	148 ± 9	< 0.0001
Systemic hematocrit [%]	31 ± 5	–	–	39 ± 4	43 ± 3	< 0.0001
Heart rate [bpm]	92 ± 19	69.3 ± 12.1	< 0.0001	69 ± 17	59.2 ± 5.3	0.001
Mean arterial pressure [mmHg]	85 ± 12	85.8 ± 8.19	0.32	80 ± 11	89.6 ± 9.6	0.0001
*ICU course and outcome*						
Full anticoagulation	23/42 (55%)	0/40 (0%)	< 0.0001	7/21 (33%)	0/33 (0%)	< 0.0001
Prophylactic anticoagulation	19/42 (45%)	0/40 (0%)	< 0.0001	14/21 (67%)	0/33 (0%)	< 0.0001
Pulmonary embolism or macro-thrombosis during ICU stay	19/39 (49%)	–	–	4/19 (21%)	–	–
ICU mortality	10/44 (23%)	–	–	7/21 (33%)	–	–
*Microcirculatory hemodynamic variables*						
Total vessel density, TVD [mm mm^−2^]	21.8 ± 5.2	19.2 ± 4.1	< 0.0001	22.6 ± 3.7	19.1 ± 4.3	< 0.0001
Functional capillary density, FCD [mm mm^−2^]	21.2 ± 4.9	18.2 ± 3.8	< 0.0001	21.6 ± 3.6	18.3 ± 4.1	< 0.0001
Red blood cell velocity, RBCv [μm s^−1^]	349 ± 41	325 ± 49	< 0.0001	336 ± 35	316 ± 52	< 0.0001
Capillary hematocrit, cHct [%]	5.17 ± 1.21	5.18 ± 0.87	< 0.0001	5.21 ± 0.81	4.62 ± 0.83	< 0.0001

Values are given as mean ± SD or median (interquartile range), as appropriate. FiO_2_, inspiratory oxygen fraction; ICU, intensive care unit; PaO_2_, arterial oxygen partial pressure; PEEP, positive end-expiratory pressure

### Measurement of the sublingual microcirculation and algorithm-based analysis

At least three HVM image sequences of four seconds duration were recorded during each measurement timepoint in all patients and volunteers according to current guidelines [11] using the CytoCam handheld incident dark field video microscope (Braedius Medical, Huizen, The Netherlands). In order to be able to perform a deep learning analysis, image sequences were obtained and treated individually. HVM image sequences were digitally recorded, cropped along the time axis, stabilized via the CCtools software (Braedius Medical, Huizen, The Netherlands) and quality graded according to strict application of Massey’s criteria [12]. They were selected for inclusion if the Massey score was below ten. The CytoCam incident dark field handheld video microscope covers a substantially larger field of view than the previous gold standard (providing a final resolution of 2208 × 1648 px versus 716 × 572 px) [13], often resulting in discarding relevant parts of this additional information in current analysis pathways. Thus, in the present study, the image sequences were split into four equally sized quadrants for analysis. The image sequences were processed and analyzed using the MicroTools advanced computer vision algorithm (Active Medical, Leiden, The Netherlands) as described in detail elsewhere [14]. In short, the algorithm employs contrast-limited adaptive histogram equalization after calculating per-pixel time-based mean values, alongside per-frame, temporal non-local means denoising technique, to prepare the image sequence data for analysis and quantification of capillary FCD, RBCv and cHct. Capillaries were defined as vessels with a diameter < 20 μm.

### Convolutional neuronal network training and validation

Image sequence data were pre-processed using the MicroTools algorithm to generate an input matrix consisting of 50.15⋅10^6^ data points for deep learning analysis. In the training dataset, the input matrix was used for supervised training of a convolutional neuronal network optimized for two-dimensional feature recognition, consisting of an iterative stack of two-dimensional convolutional, batch normalization, maximum pooling and dropout layers converging into flatten and dense layers (Additional file 1: Table S1). The convolutional neuronal network contained 12.87 × 10^6^ trainable parameters. Rectifier linear units were employed as activation functions for the convolutional and intermediate dense layers. The final dense layer was activated by a softmax function and its output interpreted as categorical data. Accuracy and loss were monitored during each training epoch via an in-training validation subset that was split at random from the training dataset with a ratio of 0.8/0.2. Model training was terminated to avoid a decrease of in-training accuracy and increase of loss, effectively avoiding overtraining. The final model parameters were recorded alongside the neuronal network structure at the end of training to obtain a reproducible deep learning-based model.

### Statistical analysis

Patient characteristics in the COVID-19 and control groups constituting the internal and external validation cohorts were reported as mean ± SD, median(IQR) or proportions, as appropriate. Comparisons between both groups were made using linear mixed model analysis with cohort status entered as fixed effects and intercepts for subjects and per-subject random slopes representing the effect on the dependent variables entered as random effects. Calculation of the deep learning and algorithm-based models was performed by treating each image sequence independently. The trained deep learning-based and algorithm-based models to identify the presence of COVID-19 disease state were validated by determining the area under the receiver operating characteristic curve in a 10^2.7^-fold bootstrap approach within both the internal and external validation datasets. Combined models were calculated via an independent 10^2.7^-fold bootstrap process and validated in the internal and external validation datasets. AUROC and 95% confidence intervals (CI) are reported as measure of performance for all models. The bootstrapped AUROC were compared between the models using two-factor linear regression analysis, with the model type and the validation cohort entered into the model as independent variables. A two-sided p-value < 0.05 was considered statistically significant. Neuronal network training and all statistical analysis were performed using the *R* environment for statistical computing, v4.1.1 [15] with the *R*-libraries Keras v2.3.0 and TensorFlow v2.2.0 [16], and Python v3.6, Keras v2.7.0 and TensorFlow v2.2.0 as backend. Further *R*-libraries used were boot v1.3–28 [17] for bootstrap analysis, pROC v1.17.0.1 [18] for receiver operating characteristic analysis and ggplot2 v2.2.1 [19] for graphical output.

## Results

**Fig. 2:**
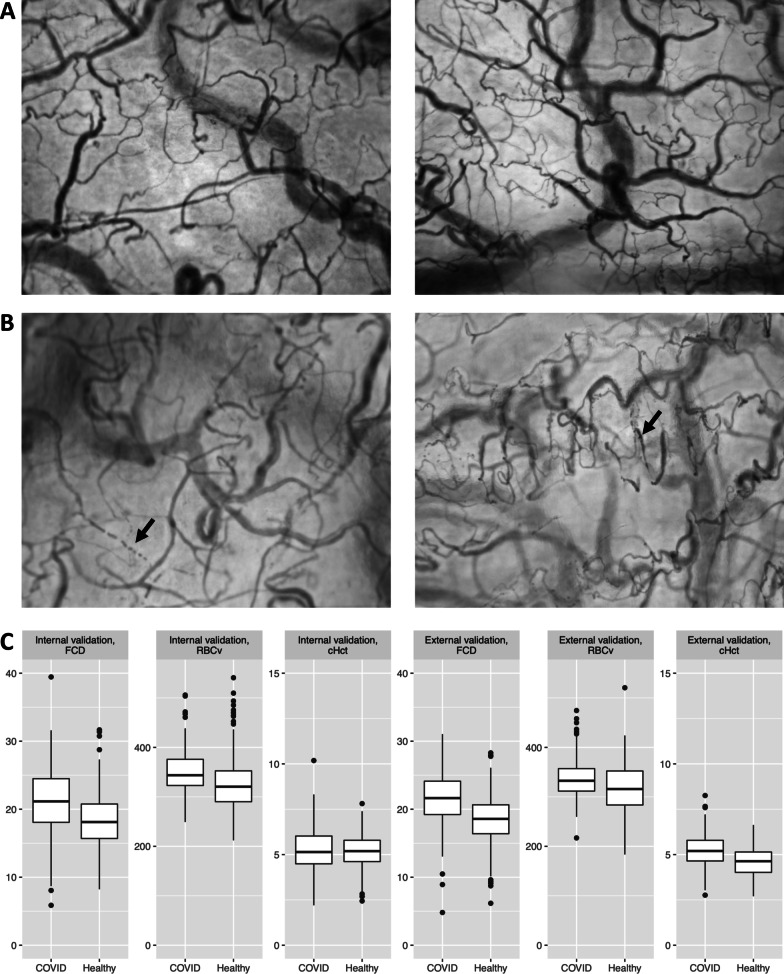
Representative examples of time-based mean images of the sublingual microcirculation obtained via handheld vital microscopy in healthy volunteers (**A**) and critically ill COVID-19 patients (**B**), and comparison of functional microcirculatory hemodynamic variables in both groups (**C**). Critically ill COVID-19 patients display disseminated intravascular coagulation (arrow, B left image) and red blood cell microaggregates (arrow, B right image) as previously described [2], representing examples for disease-specific morphological changes with respect to the healthy volunteers. FCD, functional capillary density; RBCv, red blood cell velocity; cHct, capillary hematocrit. Units are [mm mm^−2^] for FCD, [μm s^−1^] for RBCv and [%] for cHct

Six thousand ninety-two HVM image sequences of the sublingual microcirculation obtained in 157 individuals were included in the analysis. Of these, 1240 image sequences in 59 critically ill COVID-19 patients (age 59.5 ± 10.6 years, 80% male, BMI 29.0 ± 6.0 kg m^−2^) and 2476 image sequences in 40 healthy volunteers (age 24.1 ± 1.8 years, 42.5% male, BMI 22.8 ± 2.9 kg m^−2^) were included in the training and internal validation cohort. A further 1896 HVM image sequences in 25 critically ill COVID-19 patients (age 60.8 ± 10.7 years, 72%male, BMI 30.0 ± 6.0 kg m^−2^) and 480 image sequences in 33 healthy volunteers (age 23.1 ± 4.5 years, 55% male, BMI 23.1 ± 4.5 kg m^−2^) independently obtained in separate cohorts consisting of patients treated in different hospitals were included in the external validation cohort (Table 1, representative examples are shown in Fig. 2). A total of 601 image sequences were assigned a Massey score greater of equal than 10 and were not included in the study (Additional file 1: Table S2). Multiple sets of image sequences were obtained per measurement timepoint and longitudinally at different timepoints in the COVID-19 ICU cohorts, resulting in a consistent distribution of image sequences per patient and measurement timepoint across all cohorts (median 8–16, Additional file 1: Table S2). The COVID-19 patients, in contrast to the healthy volunteers, presented with a prevalence of pre-existing cardiovascular and pulmonary disease between 14 and 46%, and between 14 and 47% were regularly taking respective medication, with similar characteristics in the internal and external validation cohorts (Additional file 1: Table S3). At the time of measurement, they were mechanically ventilated, mildly hypoxemic, and 70% of them needed vasopressor support. Overall intensive care unit (ICU) mortality among the COVID-19 patients was 26% (Table 1). Congruent with previous findings [2], the critically ill COVID-19 patients’ microcirculatory hemodynamic variables indicated an elevated functional state as compared to the healthy volunteers in both the internal and the independent external validation cohorts in the present study (*P* = 0.005 for FCD, *P* < 0.0001 for RBCv and cHct, Table 1).

### Neuronal network training and validation

**Fig. 3:**
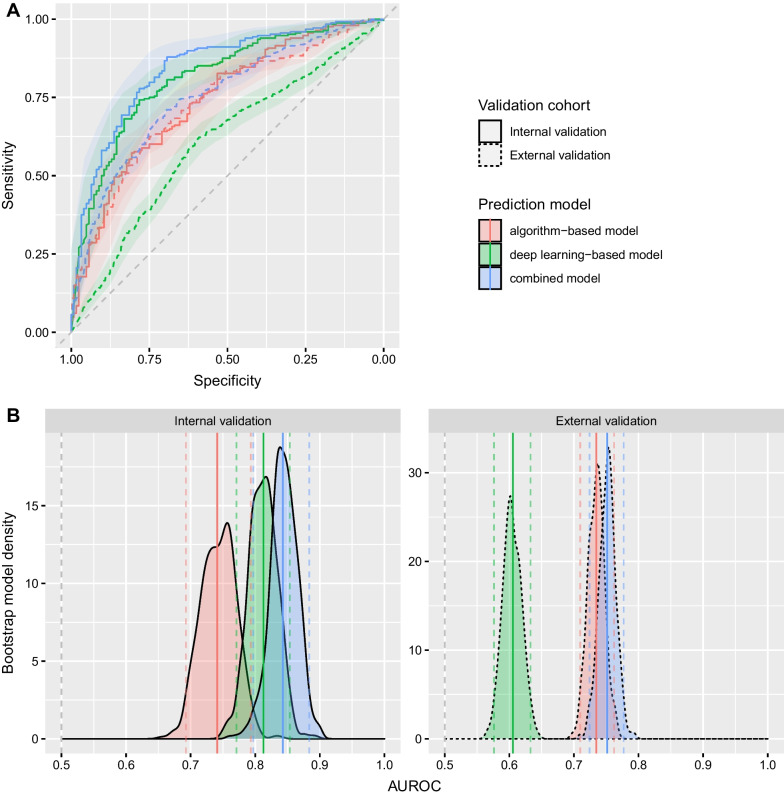
The performance of the algorithm-based, deep learning-based and combined models is demonstrated by the ROC curves for detection of COVID-19 status (**A**) and the density distributions resulting from the bootstrapped models for AUROC (**B**). Acceptable AUROC are shown even in the external validation in addition to the high sensitivity and specificity of the models in the internal validation. Dashed gray lines represent the identity line where sensitivity equals (1-specificity). Colored solid vertical lines in (**B**) represent per-model mean AUROC and colored dashed vertical lines represent the per-model 95% confidence interval of the bootstrapped models for AUROC. AUROC, bootstrap area under the receiver operating characteristic curve; ROC, receiver operating characteristic

For neuronal network training, 3344 image sequences randomly assigned to the training dataset were again randomly split into 2676 image sequences used to generate the training input matrix and 668 image sequences used for in-training validation of the neuronal network parameters (Fig. 1). During iterative training, in-training accuracy reached a peak of 0.75 after 275 training epochs without occurrence of overfitting according to in-training validation (Additional file 1: Fig. S1). The training was thereafter terminated, and the final model parameters were recorded. Bootstrapped evaluation of the fitted deep learning-based model in the internal validation dataset consisting of 372 image sequences, yielded an AUROC(CI) of 0.75 (0.69–0.79) for the identification of the presence of microcirculatory alterations associated with COVID-19 status (Fig. 3, Table 2). Bootstrapped evaluation of the same model in the external validation dataset consisting of 2260 image sequences recorded in different centers, yielded an AUROC(CI) of 0.61 (0.58–0.63).

**Table 2:** Comparison of area under the curve for identification of the presence of microcirculatory alterations associated with COVID-19 status between the algorithm-based, deep learning-based and combined models in internal and external validation

Model type	AUROC (CI) for identification of COVID-19 status		Linear regression estimates as mean difference ± S.E. (CI)		T statistic and *P* value	
	Internal validation cohort	External validation cohort	Between-model comparison	Between-cohort comparison	Between-model comparison	Between-cohort comparison
Algorithm-based model	0.74 (0.69–0.79)	0.73 (0.71–0.76)	–	− 0.10 ± 0 .00 (− 0.10 to − 0.10)	–	− 61.88, < 0.0001
Deep learning-based model	0.81 (0.76–0.86)	0.61 (0.58–0.63)	− 0.03 ± 0.00 (− 0.03 to − 0.03)		− 15.04, < 0.0001	
Combined model	0.84 (0.80–0.89)	0.75 (0.73–0.78)	0.06 ± 0.00 (0.06–0.06)		30.20, < 0.0001	

*AUROC* bootstrap area under the receiver operating characteristic curve, *CI* 95% confidence interval, *S.E.* standard error

### Combination with algorithm-based analysis and between-model comparison

In the training dataset including the training and in-training validation subsets, algorithm-based quantitative analysis of the HVM image sequences using MicroTools revealed in the healthy volunteers versus COVID-19 patients a FCD of 18.2 ± 3.8 versus 21.2 ± 5.0 mm mm^−2^, RBCv of 325 ± 49 versus 349 ± 44 μm s^−1^, and cHct of 5.18 ± 0.87 versus 5.17 ± 1.21% (Table 1, Fig. 2C). In a logistic regression model calculated from these 3344 image sequences, all three variables contributed to the model (Additional file 1: Table S4). Bootstrapped evaluation of the fitted algorithm-based model in the internal validation dataset consisting of 372 image sequences yielded an AUROC(CI) of 0.74 (0.69–0.79) for identification of the presence of microcirculatory alterations associated with COVID-19 status (Fig. 3, Table 2). A combined logistic regression model calculated via a bootstrap process within the internal validation dataset, based on the COVID-19 status anticipated by the deep learning and algorithm-based models, yielded in the internal validation an AUROC(CI) of 0.84 (0.80–0.89) for identification of the presence of microcirculatory alterations associated with COVID-19 status. The external validation dataset, similarly to the internal validation dataset, also displayed an increased functional microcirculatory hemodynamic status in the COVID-19 patients as compared to healthy volunteers (Table 1, Fig. 2C). Applied to the external validation dataset via bootstrapping, the algorithm-based and combined models both displayed lower performance in comparison to the internal validation (*P* < 0.0001), but sensitivity and specificity were maintained above 70% with the algorithm-based model AUROC(CI) at 0.73 (0.71–0.76), and the combined model at 0.75 (0.73–0.78). Comparison of the deep learning-based, algorithm-based, and combined models demonstrated a mean difference (CI) in AUROC of − 0.03 (0.03–0.03) and 0.06 (0.06–0.06) for the deep learning-based and combined model (*P* < 0.0001).

## Discussion

The present study demonstrates the successful training of a two-dimensional convolutional neuronal network to differentiate critically ill COVID-19 patients from healthy volunteers using HVM image sequences of the sublingual microcirculation. The deep learning-based model performed better that a model built from the main determinants of microcirculatory function determined using the MicroTools algorithm to recognize the COVID-19 patients in internal validation, which was reversed in external validation. In both cases, a combination of the deep learning-based with the algorithm-determined functional hemodynamic measurements, achieved the best performance to detect COVID-19 patients in measurements of the sublingual microcirculation.

### Handheld vital microscopy image sequences as basis for neuronal network training

Deep learning and other machine learning methodology has shown encouraging results in interpreting the large amount of commonly collected clinical data in an ICU environment to provide decision support [20]. However, new measurement methods must complement this optimized combination of clinical data, to further increase the useful information emerging from such endeavors and provide deeper insight into the physiological processes associated with critical illness [21]. In the present study, we successfully applied deep learning methodology to dark field microscopy image sequences, a measurement method that has previously been shown to yield detailed information not only on the most important determinants of oxygen delivery to the tissues, but also on changes effected by disease processes that alter the morphology of the microvessels, the red blood cell configurations, and the movement patterns contained within them [1, 3, 4, 22]. The use of image sequences of the sublingual microcirculation in a deep learning-based approach expands on previous studies employing neuronal networks to detect abnormalities such as the diabetic changes [23] or changes related to other cardiovascular risk factors [7], in the retinal microvessels. The present study adds two new perspectives. First, the successful recognition of COVID-19 patients demonstrates the viability of a deep learning-based approach to gain information on the state of the systemic microcirculation that may be relevant for the diagnosis of a specific disease state. Second, by showing that the combination of deep learning-based analysis and algorithm-based quantification of functional microcirculatory hemodynamic variables is superior to either method, it may pave the way toward a better understanding of disease-associated changes in the sublingual microcirculation.

### Improved identification of microcirculatory alterations associated with disease state through combined functional and disease-specific assessment

Previous attempts at using deep learning-based approaches to detect COVID-19 disease state have mainly focused on the analysis of chest x-ray and computed tomography data. Recent studies employing various deep learning techniques have been described to reach a sensitivity and specificity of 60–90% [24–26], with values in the higher end of the range for computed tomography [27, 28], while nucleic acid amplification tests in nasopharyngeal swabs, which are regarded as the clinical gold standard for diagnosis of SARS-CoV-2 infection, have previously reported sensitivities between 60 and 95% alongside a specificity > 95% in the presence of symptoms for later positivity [29]. The use of the sublingual microcirculation to obtain diagnostic information on the presence of a specific disease, as opposed to organ-specific imaging or specific tests to detect the presence of antigens, provides the unique opportunity to assess the state of functional physiological adaptation mechanisms alongside disease-specific features. In critically ill COVID-19 patients, such specific morphological features could include disseminated intravascular coagulation and red blood cell microaggregates as previously identified [2] and shown in Fig. 2. The function of the sublingual microcirculation, on the other hand, directly reflects the oxygen delivery capacity to the tissue, and has previously been shown to tightly correlate with the outcome in critically ill patients [30, 31]. The increased microcirculatory functional capacity found in the present study is consistent with previous findings [2, 32, 33] and has been shown to persist until the occurrence multi-organ failure [2, 32] or severe endothelial dysfunction [34, 35]. However, in contrast to previous measurement methods of microcirculatory function such as subjective classification of image sequences and assignment of mean flow index (MFI) categories, the quantitative, fully automated, algorithm-based image sequence analysis enabled by MicroTools and used in the present study, allowed for accurate quantification of the functional microcirculatory hemodynamic variables, as well as the elimination of inter-observer bias introduced by subjective grading [3]. At the same time, RBCv as measured by Microtools has previously been demonstrated to differentiate all relevant MFI categories [3]. Our results demonstrate that the combination of both functional and morphological feature detection enables the differentiation of image sequences recorded in critically ill COVID-19 patients and healthy volunteers, and that these noninvasive recordings of the sublingual microcirculation allow such differentiation in a manner comparable to that of chest x-ray, computed tomography, and even nucleic acid amplification tests. The comparison of critically ill COVID-19 patients and healthy volunteers as employed in the present study, however, does not differentiate between changes specific to COVID-19 and changes induced by critical illness in general. As a first study employing this methodology, the results obtained encourage the application of deep learning-based methods to the analysis of the sublingual microcirculation for comparison of different cohorts of critically ill patients, for example, critically ill COVID-19 patients and septic patients, and to expand the methodology not only for diagnostic purposes, but also to guide therapy as previously suggested for the algorithm-based approach [1, 4]. Further, the finding that the deep learning-based model demonstrated a higher performance than the algorithm-based model in the internal validation dataset originating from the same cohort as the training dataset, but a lower performance in the external validation dataset, implies as expected that the characteristics identified by the neuronal network may be less generalizable than the functional adaptation. At the same time, this finding underscores the remarkable robustness of the algorithm-based analysis also in external validation. The combination of both methodologies in the present study thus contributed to a mitigation, resulting in a model with high sensitivity and specificity. In the future, further refined neuronal networks and adapted pathways for data pre-processing, alongside new developments in measurement methodology such as multi-wavelength microscopes [36], may help to increase the generalizability of the deep learning approach to the analysis of the sublingual microcirculation.

### Limitations

The present study has several limitations. First, while both internal and external validation of a deep learning model to identify the presence of microcirculatory alterations associated with COVID-19 status in the sublingual microcirculation were demonstrated in critically ill COVID-19 patients and healthy volunteers, further studies are needed to discern characteristics associated with critical illness in general as opposed to COVID-19 specific abnormalities. The generalizability of the current findings is strengthened by using an international, multi-central dataset originating from ICUs with different treatment algorithms and measurements obtained by different researchers, and by the collection of the measurements before the emergence of virus variants of concern with markedly different manifestation of critical illness [10, 37] and major changes in treatment regimens [10, 38]. The bootstrapped models further increase the generalizability of the analysis within the respective datasets [17]. Also, the algorithm-based pre-processing of the HVM image sequences for use in the convolutional neuronal network has largely mitigated potential technical differences such as in contrasting or illumination between the different datasets, focusing the remaining distinctions between the datasets on differences that could result from variation in patient population or ICU management. Second, the algorithm-based pre-processing of HVM image sequence data for the application in neuronal network training and the neuronal network structure as applied in the present study can be subjected to further development, namely, to include additional aspects of microcirculatory function. The present study, by combining the results from a neuronal network with algorithm-based functional microcirculatory hemodynamic assessments, emphasizes the promise associated with such developments. Lastly, due to treating each image sequence independently, the association of each image sequence to an individual patient or measurement timepoint is not taken into account, representing a potential source of bias. However, as shown in Additional file 1: Table S2, a consistent distribution of image sequences per patient and per measurement timepoint across all cohorts included in the present study, and also of absolute image sequences per patient within the training and internal validation, and the external validation datasets, ensures to minimize such potential bias.

## Conclusion

In conclusion, the present study demonstrated the successful use of a convolutional neuronal network to derive a deep learning-based model differentiating critically ill COVID-19 patients from heathy volunteers in HVM image sequences recorded sublingually. The combination with an algorithm-based approach to quantify the functional state of the microcirculation markedly increased the sensitivity and specificity as compared to either approach alone and enabled successful internal and external validation of the identification of the presence of microcirculatory alterations associated with COVID-19 status. Further studies are warranted to expand these findings to other etiologies of critical illness.

## Supplementary Information

**Additional file 1**. Supplementary information.

## Data Availability

The datasets that support the conclusions of this article are available from the corresponding author on reasonable request.
