# Supplementary material for: Microcirculatory alterations in critically ill COVID-19 patients analyzed using artificial intelligence
Source: Crit Care. 2022 Oct 14;26:311. doi: 10.1186/s13054-022-04190-y (PMC9568900; doi:10.1186/s13054-022-04190-y)

Supplementary Information

Microcirculatory alterations in critically ill COVID-19 patients analyzed using artificial intelligence

Matthias Peter HILTY, Emanuele FAVARON, Pedro David WENDEL GARCIA, Yavuz AHISKA, Zuhre UZ, Sakir AKIN, Moritz FLICK, Sesmu ARBOUS, Daniel A HOFMAENNER, Bernd SAUGEL, Henrik ENDEMAN, Reto Andreas SCHUEPBACH, Can INCE

Supplementary Table S1: Composition of the neuronal network used for identification of the presence of microcirculatory alterations associated with COVID-19 disease state 2

Supplementary Table S2: Distribution of image sequences across patients and measurement timepoints in the COVID-19 ICU cohorts, implementation of quality criteria 2

Supplementary Table S3: Additional physiological status, comorbidities and previous medication of the COVID-19 patients and healthy volunteers at study inclusion 3

Supplementary Table S4: Logistic regression model for identification of the presence of microcirculatory alterations associated with COVID-19 state 4

Supplementary Figure S1: Training and in-training validation of the two-dimensional convolutional neuronal network for identification of the presence of microcirculatory alterations associated with COVID-19 disease state 4

Supplementary Table S1: Composition of the neuronal network used for identification of the presence of microcirculatory alterations associated with COVID-19 disease state

Table S1. Composition of the neuronal network used for identification of the presence of microcirculatory alterations associated with COVID-19 disease state. Connections between layers are implemented from top to bottom. The output size of each layer corresponds to the input size of the consecutive layer. The final neuronal network consisted of $12.87 \cdot 10^6$ trainable parameters. RELU, rectifier linear unit; 2D, two-dimensional.

layer type	*kernel size / pool size / units*	*activation*	*attributes*
**input** -> 2D conv	3, 3	RELU	32 filters
batch normalization			
2D max pooling	3, 3		
dropout			rate = 0.25
2D conv	3, 3	RELU	64 filters
batch normalization			
2D conv	3, 3	RELU	64 filters
batch normalization			
2D max pooling	2, 2		
dropout			rate = 0.25
2D conv	3, 3	RELU	128 filters
batch normalization			
2D conv	3, 3	RELU	128 filters
batch normalization			
2D max pooling	2, 2		
dropout			rate = 0.25
flatten			
dense	1024 units	RELU	
batch normalization			
dropout			rate = 0.5
dense -> **output**	2 units	softmax	

Supplementary Table S2: Distribution of image sequences across patients and measurement timepoints in the COVID-19 ICU cohorts, implementation of quality criteria

Table S2. The different cohorts of critically ill COVID-19 patients included in the present study contained similar numbers of image sequences per patient and per timepoint. ICU, intensive care unit; IQR, interquartile range.

COVID-19 ICU cohorts	*Number of patients; n*	*Number of image sequences included in the study; n*	*Number of measurement timepoints; n*	*Image sequences per patient per timepoint; median (IQR)*	*Number of image sequences with Massey score ≥10; n*
University Hospital of Zurich, Zurich, Switzerland	23	355	5	12 (12 - 12)	105
Erasmus Medical Center, Rotterdam, The Netherlands	22	664	9	8 (4 - 8)	128
Haga Hospital, Den Hague, The Netherlands	14	220	1	16 (12 - 16)	32
Leiden Medical Center, Leiden, The Netherlands	25	1896	11	12 (8 - 16)	116
Volunteer cohorts					
University Medical Center Hamburg-Eppendorf, Hamburg, Germany	40	2476	-	-	84
University Hospital of Zurich, Zurich, Switzerland	33	480	-	-	136

Supplementary Table S3: Additional physiological status, comorbidities and previous medication of the COVID-19 patients and healthy volunteers at study inclusion

Table S3. Additional physiological status, comorbidities and previous medication of the critically ill COVID-19 patients at study inclusion, in the training and internal validation, and the external validation cohorts.

	Training and internal validation cohort			*External validation cohort*		
	COVID-19 patients (Zurich / Rotterdam / The Hague cohorts) n = 59	*Healthy volunteers (Hamburg cohort) n = 40*	*p value*	*COVID-19 patients (Leiden cohort) n = 25*	*Healthy volunteers (Zurich cohort) n = 33*	*p value*
Additional physiological status at study inclusion						
Mean central venous pressure [mmHg]	9.7 ± 5.3	-	-	9.2 ± 4.4	-	-
Fluid Balance [ml] collected in the last 24h	312 ± 1001	-	-	1636 ± 2843	-	-
Norepinephrine administration at any dose	36/53 (69%)	0/40 (0%)	< 0.0001	15/21 (71%)	0/33 (0%)	< 0.0001
Epinephrine/Dobutamine/Milrinone/other vasoactive drugs	0/42 (0%)	0/40 (0%)	< 0.0001	1/21 (5%)	0/33 (0%)	< 0.0001
Mean Norepinephrine Dose [µg/kg/min]	0.1 ± 0.13	0 ± 0	< 0.0001	0.04 ± 0.07	0 ± 0	< 0.0001
Mean Heparin Dose [IE/24h]	34’606 ± 18’429	0 ± 0	< 0.0001	24’275 ± 6’735	0 ± 0	< 0.0001
Temperature [°C]	37.5 ± 1	-	-	36.6 ± 0.7	-	-
SOFA Score	8 ± 4	-	-	8 ± 2	-	-
C-reactive protein [mg L^{-1}]	163 (100-285)	-	-	19 (6-53)	-	-
Procalcitonin [µg L^{-1}]	0.92 (0.36-3.6)	-	-	-	-	-
Interleukin-6 [pg mL^{-1}]	92.3 (49-211)	-	-	-	-	-
White blood cells [10^9 L^{-1}]	12.9 ± 5.7	-	-	11.3 ± 4	-	-
Neutrophil granulocytes [10^9 L^{-1}]	9.6 ± 4.6	-	-	-	-	-
Lymphocytes [10^9 L^{-1}]	1.2 (0.9-1.7)	-	-	0.7 (0.5-0.9)	-	-
Neutrophil/Lymphocyte ratio	7.2 (5.3-10)	-	-	-	-	-
D-dimers [mg L^{-1}]	2.4 (1.2-5)	-	-	1.9 (0.8-8.5)	-	-
Lactate dehydrogenase [U L^{-1}]	374 (304-514)	-	-	-	-	-
eGFR [mL min^{-1} $1.73m^{-2}$]	62 (34-87)	-	-	-	-	-
Comorbidities						
- arterial hypertension	18/39 (46%)	-	-	9/21 (43%)	-	-
- diabetes mellitus	19/39 (49%)	-	-	1/21 (5%)	-	-
- chronic heart disease	13/53 (25%)	-	-	0/25 (0%)	-	-
- chronic pulmonary disease	21/53 (40%)	-	-	3/21 (14 %)	-	-
- chronic kidney disease	4/53 (8%)	-	-	0/25 (0%)	-	-
- chronic liver disease	2/53 (4%)	-	-	0/25 (0%)	-	-
- immunodeficiency	7/53 (13%)	-	-	0/25 (0%)	-	-
Previous medication						
- angiotensin converting enzyme inhibitors	3/44 (7%)	-	-	0/25 (0%)	-	-
- other anti-hypertensive agents	19/43 (44%)	-	-	9/21 (43%)	-	-
- anti-coagulants	2/43 (5%)	-	-	3/21 (14%)	-	-
- anti-aggregatory agents	3/43 (7%)	-	-	0/25 (0%)	-	-
- bronchodilators	8/43 (19%)	-	-	3/21 (14%)	-	-
- immunosuppressant agents	7/43 (16%)	-	-	3/21 (14 %)	-	-

Supplementary Table S4: Logistic regression model for identification of the presence of microcirculatory alterations associated with COVID-19 state

Table S4. Logistic regression model for identification of the presence of microcirculatory alterations associated with COVID-19 state, derived from the training dataset using the algorithm-based quantitative parameters of microcirculatory function derived from the sublingual microcirculation image sequences. CI, 95 % confidence interval; S.E., standard error; FCD, functional capillary density; RBCv, red blood cell velocity

Quantitative parameter of microcirculatory function	*Odds ratio ± S.E. (CI)*	*z statistic*	*P value*
FCD [mm mm^{-2}]	0.81 ± 1.01 (0.79 - 0.83)	-17.94	< 0.0001
RBCv [μm s^{-1}]	0.99 ± 1.00 (0.99 - 0.99)	-11.42	< 0.0001
cHct [1]	$3.96 \cdot 10^{11} \pm 1.81 \cdot 10^{2}$ ($1.54 \cdot 10^{8} - 1.1 \cdot 10^{17}$)	5.13	< 0.0001

Supplementary Figure S1: Training and in-training validation of the two-dimensional convolutional neuronal network for identification of the presence of microcirculatory alterations associated with COVID-19 disease state

Figure S1. Training and in-training validation of the two-dimensional convolutional neuronal network for identification of the presence of microcirculatory alterations associated with COVID-19 disease state. The accuracy and loss show convergence across the training epochs without signs of overtraining. The learning algorithm shows progressive increase in accuracy of classification within the in-training validation data, with a decrease of loss of redundant data.

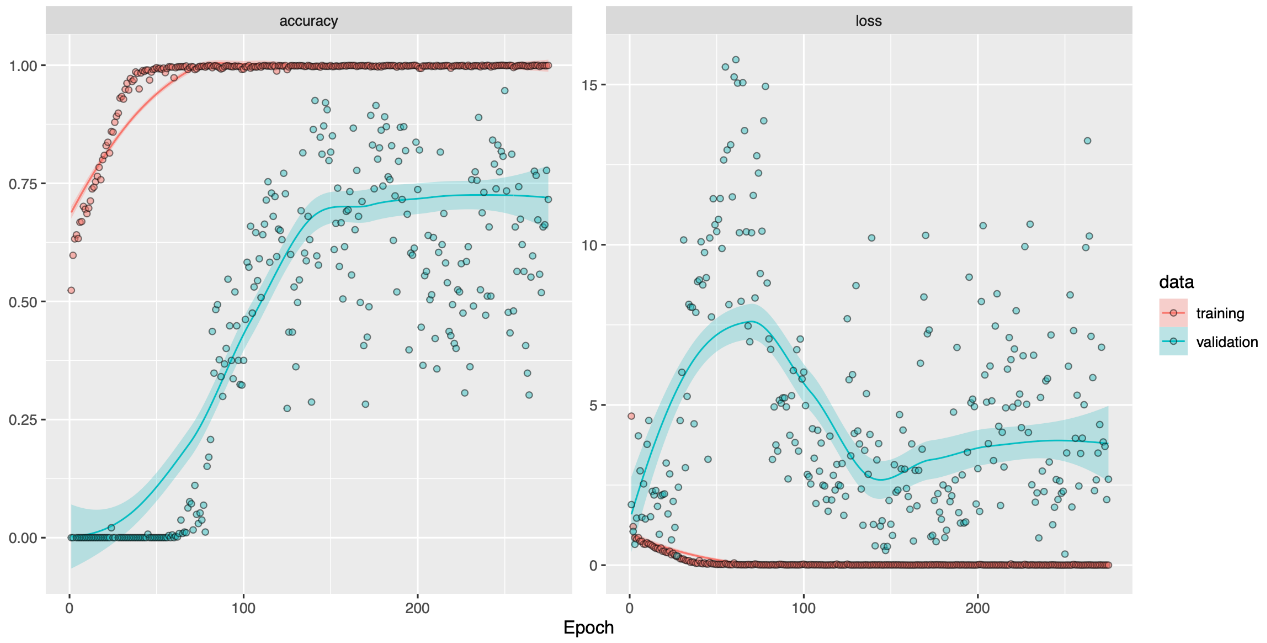

Supplement: Supplementary file 1 — Additional file 1. Supplementary information. [file 13054_2022_4190_MOESM1_ESM.pdf]
